# Bilateral oedipism: a case of extreme self-harm in an African society averse to self-mutilation

**DOI:** 10.4314/ahs.v20i4.37

**Published:** 2020-12

**Authors:** Ugochukwu Uzodimma Nnadozie, Okwudili Nicodemus Obayi, Edak Ezeanosike, Christian Eze, Charles Chidiebele Maduba, Fortune Udoka Nnadozie, Kene Joe-Akunne, Christian Chidebe Anikwe

**Affiliations:** 1 Department of Surgery, Ebonyi State University Abakaliki, Ebonyi State Nigeria; 2 Department of Psyciatry, Ebonyi State University Abakaliki, Ebonyi State Nigeria; 3 Department of Ophthalmology, Ebonyi State University Abakaliki, Ebonyi State Nigeria; 4 Division of Plastic Surgery, Department of Surgery, Alex Ekwueme Federal University Teaching Hospital Abakaliki, Ebonyi State, Nigeria; 5 Department of Psychiatry, Federal Medical Center Umuahia, Abia State. Nigeria; 6 Department of Ophthalmology, Alex Ekwueme Federal University Teaching Hospital Abakaliki, Ebonyi State, Nigeria; 7 Department of Obstetrics and Gynaecology, Alex Ekwueme Federal University Teaching Hospital Abakaliki, Ebonyi State, Nigeria

## Introduction

Autoenucleation, an unusual form of extreme self-harm, first appeared in scientific literature in 1846, reported by Bergman but later surnamed Oedipism by Blonel in 1906, after a major character portrayed in Sophocles's play – Oedipus Rex.^1^ Oedipus was the king of Thebes who unwittingly killed his father and married his own mother. The guilt of patricide and incest moved him to gorge out his eyes to atone for his mortal sins.

The prevalence of autoenucleation is not certain as various rates have been reported, though most reports and studies have shown that there is equal male to female ratio and age range of victims being usually between 15 and 53 years.^2^ Most autoenucleations were performed with just bare fingers,^3^ though hooks, nails, hangers, spoons, scissors and knives were also instruments reportedly used.

Besides psychiatric illnesses and organic/neurological conditions, self-enucleation is also at times associated with alcohol/drug-related disorders.^4^ Most patients have been reported to suffer from acute or chronic psychoses with chronic schizophrenia being the commonest association.^5^

Several fatal complications have been described in the event of autoenucleation, such as blood loss^3^, suppurative meningitis,^6^ and cerebrospinal fluid leak and pituitary failure,^7^ among others. Autoenucleation, like other forms of self-mutilation, is not acceptable in most societies of the world. In a community where there is high stigma towards people with mental illness or where some believe that mental disorder is a punishment for the ‘evil’ behaviour of the victim, orthodox care is hardly sought and if at all, often very late. The situation is worsened when an act seen as a taboo by the people, such as self-enucleation, is carried out by the already-condemned mentally-ill.

## Case report

A 24-year old farmer from a neighbouring State in SouthEast Nigeria presented at the Accident and Emergency department of our hospital (Alex Ekwueme Federal University Teaching Hospital Abakaliki) a week after a digital bilateral self-enucleation. He was accompanied by his older brother who narrated that when he entered patient's room, he found him with blood on his face, attempting to rip off both ears with his bare hands while his eyeballs were found lying on the floor.

Patient was reported to have been exhibiting some abnormal behaviours over the preceding two years, characterized mainly by verbal and physical aggression (especially towards family members), suspiciousness, belief that events around were referring to him, belief that his actions were controlled by external agencies, and occasional fearfulness that at times warranted his fleeing from the house even in the night to hide in the nearby bush. There was a positive history of heavy and long-standing daily use of cannabis and alcohol.

Parents sought relief from herbal medication which he objected strongly to and was therefore taken to a prayer house for healing. He was very bitter with the family for taking him to a prayer house without his consent. He was particularly angry with the father whom he believed disliked him, had been unnecessarily critical of his behaviour, and was instrumental to his being taken to a prayer house. He absconded from the prayer house after two days of prayer and back home he threatened to harm anybody that came his way.

**Figure 1:**
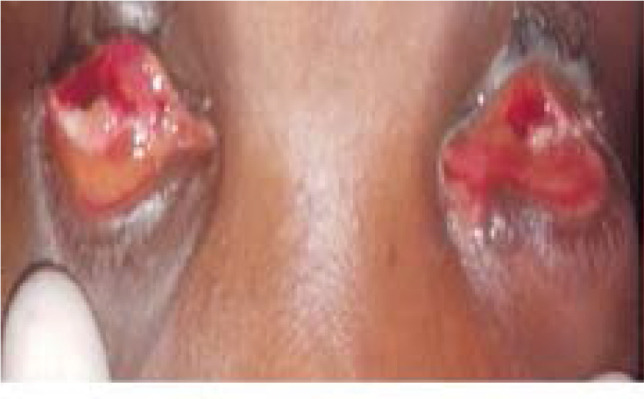
Both eyes gouged.

He reported that a thought came to him that plucking out his eyes would bring relief to his problems. Hence, he yielded to the thought and gouged out his eyes (Figure 1) with his fingers in an attempt to ease some perceived discomfort.

**Figure 2:**
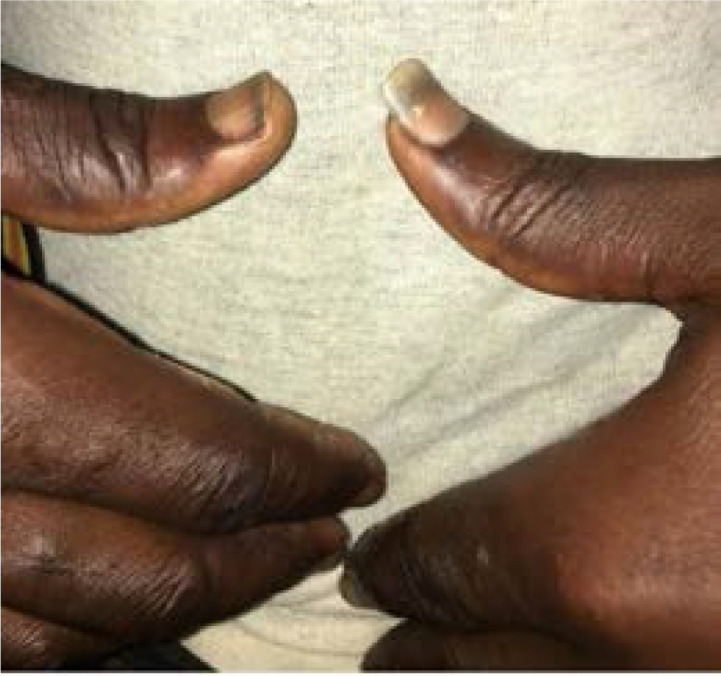
shows his prominent thumb fingernails he used in the act

Figure 2 shows his prominent thumb fingernails he used perceived discomfort in the act. A B-scan ultrasonography was carried out which showed echogenic collection and absent globes within the eye socket. This extreme self-mutilating thought, he said, came immediately after he returned from the prayer house and continued to re-occur until he obeyed it by the third day of his return from the prayer house.

He described the autoenucleation procedure as painless and easy, though he encountered some difficulty in completing the enucleation in the left eye, but after some maneuver, he succeeded. There was a little blood loss estimated to be about 10mls. He subsequently developed mild lid swellings.

He was brought to the hospital by the family members to explore the possibility of an eye transplant. Ocular examination revealed empty sockets with redundant haemorrhagic conjunctiva. Optic nerve stump was visible in the left socket where lower lid oedema, ecchymosis and 1cm medial marginal laceration involving the canaliculus were also prominent. A diagnosis of bilateral oedipism in a patient with paranoid schizophrenia (ICD-10, code F20.0) was made and patient was commenced on antipsychotic medication.

He was also commenced on topical and systemic antibiotics plus anti-inflammatory agents. He was offered secondary orbital implants followed by fitting of custom ocular prosthesis for cosmetic rehabilitation. The family on discovering that their expectation of restoration of sight was not possible declined all other treatment options and he was subsequently lost to follow up.

## Discussion

This case illustrates a rare complication of psychiatric disorders. Our patient was of the male gender and in his 20s. A review of self-inflicted eye injuries by Patton (2004) showed that majority of the victims were young-to-early middle-aged males.^5^ Our patient carried out the self-enucleation with his own hands, just as in most reported cases. Unilateral self-enucleation is commoner. Hence, ours is one of the relatively rare cases of bilateral self-enucleation.^8^ Against the initial traditional belief that self-enucleation was as a result of psycho-sexual conflicts, including those arising from Freud's Oedipal complex and Christian religious teaching, the case reported here was due to untreated psychotic illness as later knowledge has proved.^9^ Only a little quantity of blood was lost in the process. When enucleation is complete, the process is less painful than partial enucleation possibly because of the natural plane in the socket. This is in keeping with the earlier noted fact that complete digital self-enucleation remarkably tends to cause little haemorrhage and patients' pain sensation markedly reduced.^10^

The case presented shares some semblance with previously published reports. Foremost, it lends credence to the possibility of bilateral self-enucleation as noted in another report. 8 Though the patient reported here had a psychotic illness, he was also known to have been a chronic user of psychoactive substances – a known singular factor to self-mutilation.^4^

## Conclusion

Bilateral oedipism is a rare occurrence, especially in the Igbo society of Africa where self-mutilation is generally aversed to irrespective of cause. Mental health awareness, early treatment of persons with mental disorders can prevent this extreme self-harm.
